# Dynamic ^31^P-MRI and ^31^P-MRS of lower leg muscles in heart failure patients

**DOI:** 10.1038/s41598-021-86392-y

**Published:** 2021-04-01

**Authors:** Rajiv G. Menon, Ding Xia, Stuart D. Katz, Ravinder R. Regatte

**Affiliations:** 1 Bernard and Irene Schwartz Center for Biomedical Imaging, New York University Langone Medical Center, 660 1st Ave, 4th Floor, New York, NY 10016 USA; 2 Division of Cardiology, Department of Medicine, New York University Langone Medical Center, New York, NY USA

**Keywords:** Magnetic resonance imaging, Diagnostic markers

## Abstract

Impaired oxidative metabolism is one of multi-variate factors leading to exercise intolerance in heart failure patients. The purpose of the study was to demonstrate the use of dynamic ^31^P magnetic resonance spectroscopy (MRS) and ^31^P magnetic resonance imaging (MRI) techniques to measure PCr resynthesis rate post-exercise as a biomarker for oxidative metabolism in skeletal muscle in HF patients and controls. In this prospective imaging study, we recruited six HF patients and five healthy controls. The imaging protocol included ^31^P-MRS, spectrally selective 3D turbo spin echo for ^31^P-MRI, and Dixon multi-echo GRE for fat–water imaging on a 3 T clinical MRI scanner. All the subjects were scanned pre-exercise, during plantar flexion exercise, and post-exercise recovery, with two rounds of exercise for ^31^P -MRS and ^31^P-MRI, respectively. Unpaired t-tests were used to compare ^31^P-MRS and ^31^P-MRI results between the HF and control cohorts. The results show that PCr resynthesis rate was significantly slower in the HF cohort compared to the controls using ^31^P-MRS (*P* = 0.0003) and ^31^P-MRI (*P* = 0.0014). ^31^P-MRI showed significant differences between the cohorts in muscle groups (soleus (*P* = 0.0018), gastrocnemius lateral (*P* = 0.0007) and gastrocnemius medial (*P* = 0.0054)). The results from this study suggest that ^31^P-MRS/^31^P-MRI may be used to quantify lower leg muscle oxidative metabolism in HF patients, with ^31^P-MRI giving an additional advantage of allowing further localization of oxidative metabolism deficits. Upon further validation, these techniques may serve as a potentially useful clinical imaging biomarker for staging and monitoring therapies in HF-patients.

## Introduction

Heart failure (HF) is characterized by the inability of the heart to deliver adequate oxygen and nutrients to peripheral tissues that subsequently has systemic effects on multiple organs^1,2^. One of the classic hallmarks of HF includes exercise intolerance (EI) and fatigue in skeletal muscle^3^. While reduced cardiac output is one of the primary reasons for reduced peak exercise capacity, it is not the sole determinant. Downstream effects of reduced cardiac output bring about bioenergetic changes, perfusion deficits, and skeletal muscle adaptations result in reduced peak exercise capacity^4–6^. Indeed, studies show that EI is observed for several months after cardiac transplantation and normalization of cardiac output^4,5^. Additionally, exercise training in HF patients improves exercise tolerance without improving exercise cardiac output^7^. These factors suggest that EI results from a complex multi-factorial interplay of cardiac and extra-cardiac factors that reduce oxygen utilization in metabolically active tissues^6,8,9^.

Adenosine tri-phosphate (ATP) is the primary energy currency for normal skeletal muscle contractions. Impaired oxidative metabolism that results in reduced ATP utilization causes bioenergetic adaptations in skeletal muscle and is implicated in HF development and progression. While impaired ATP metabolism plays a crucial role in other pathologies such as metabolic myopathies^10,11^ diabetes^12^ and muscular dystrophies^13^, the role of impaired oxidative metabolism in HF is not well characterized.

Phosphorus (^31^P) MRS techniques provide a unique non-invasive window into oxidative metabolism by characterizing high energy phosphates, ATP and creatine phosphate (PCr) in skeletal muscle^14,15^. Measurement of PCr resynthesis following exercise has been well reported and is a reliable measure of mitochondrial oxidative metabolism^16,17^. A number of studies have reported PCr resynthesis measurements following exercise using ^31^P-MRS^18–20^. While MRS techniques have the advantage of measuring multiple metabolites during the experiment, drawbacks of many of these studies include the usage of surface coils with limited depth sensitivity. Additionally, it is important to perform these exercise studies with low-intensity to prevent lactic acid buildup, and subsequent pH changes, which in turn increases the duration of acquisition.

^31^P imaging was first shown by Ernst, et al.^21^. ^31^P imaging methods to measure PCr resynthesis rate following exercise provides additional information and can provide new insights to the mechanistic processes underlying muscle specific pathology. Imaging with spectrally selective sequences have shown that imaging a single metabolite improves the acquisition speed. Such methods have increasingly been used to study individual metabolites^22,23^. Parasoglou, et al. showed that the PCr resynthesis rate can be reliably quantified following exercise using spectrally selective sequences^24,25^.

The goal of this study was to use conventionally used ^31^P-MRS and the 3D turbo spin echo (TSE) based spectrally selective ^31^P imaging to quantify the PCr resysnthesis rate of lower leg muscles following plantar-flexion calf exercise to assess oxidative metabolism in HF patients and control subjects. Another goal of this study was to show the complementary information obtained using PCr imaging in comparison to ^31^P-MRS.

## Results

**Figure 1:**
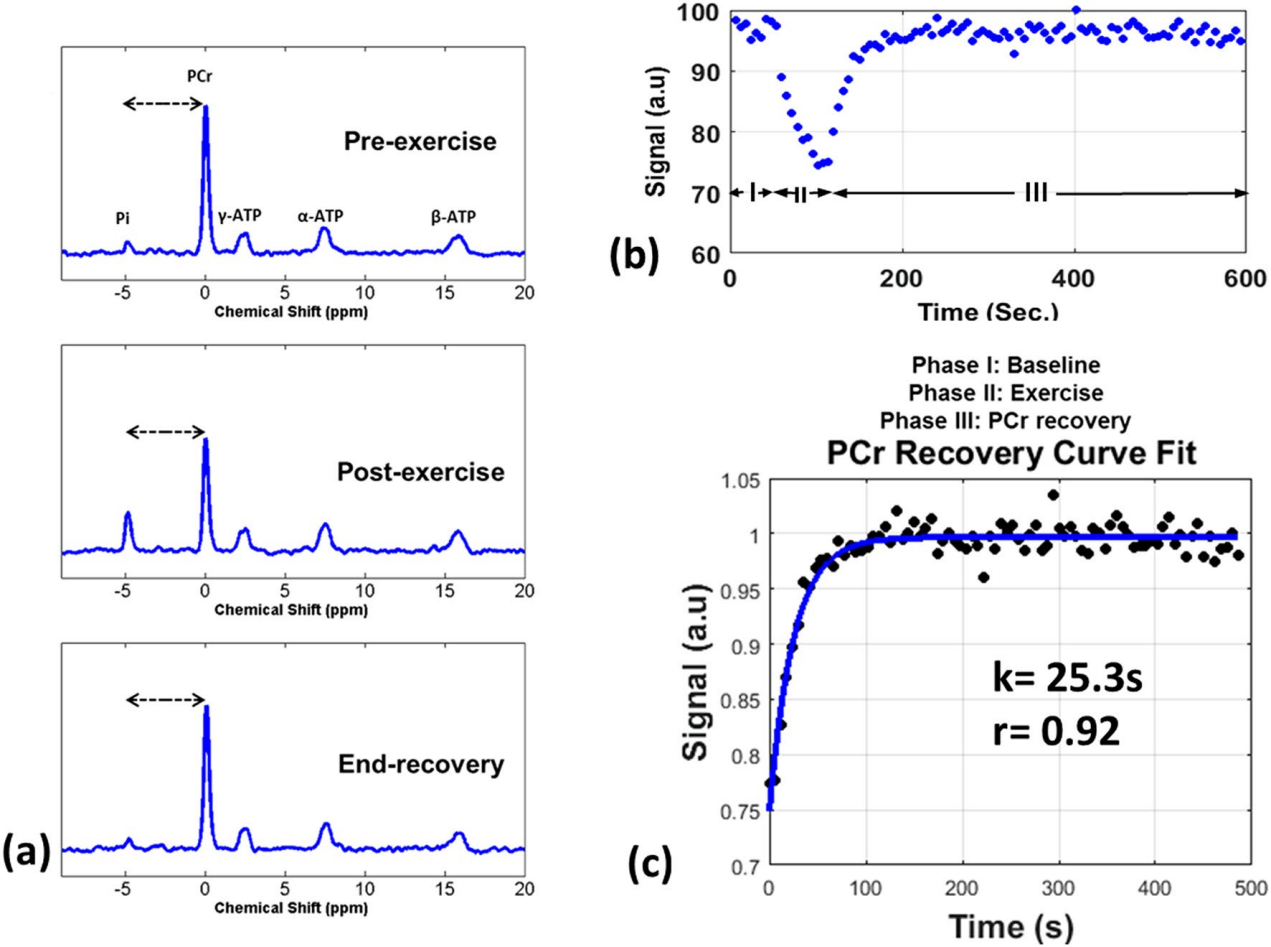
Dynamic ^31^P-MRS results in healthy volunteer. (**a**) Shows ^31^P-MR spectra from a representative healthy volunteer’s calf muscle pre-exercise, post-exercise, and at end-recovery. The dashed arrows indicate the distance between Pi and PCr peaks (**b**) shows the kinetics of PCr signal intensity during the three phases for the healthy volunteer and (**c**) shows the PCr recovery time constant (k = 25.3 s for this volunteer) after fitting the phase III PCr recovery data.

The results obtained from representative spectra using ^31^P-MRS in healthy volunteers are shown in Fig. 1. Figure 1a shows ^31^P-MRS spectra from the calf muscle pre-exercise, post-exercise and at the end of the 10 min recovery. The spectra show the depletion of PCr post-exercise, and the resynthesis of PCr at end-recovery. The pH, measured as the distance between the Pi and PCr peaks, remained constant indicating negligible pH change as a result of the exercise. Figure 1b shows plots of the PCr amplitude across the baseline (phase I), exercise (phase II), and recovery phases (phase III). Figure 1c shows data fit according to Eq. 1 for the post-exercise recovery phase III for this subject. The PCr recovery rate constant across all control subjects was 26.73 ± 4.49 s (mean ± standard deviation).

**Figure 2:**
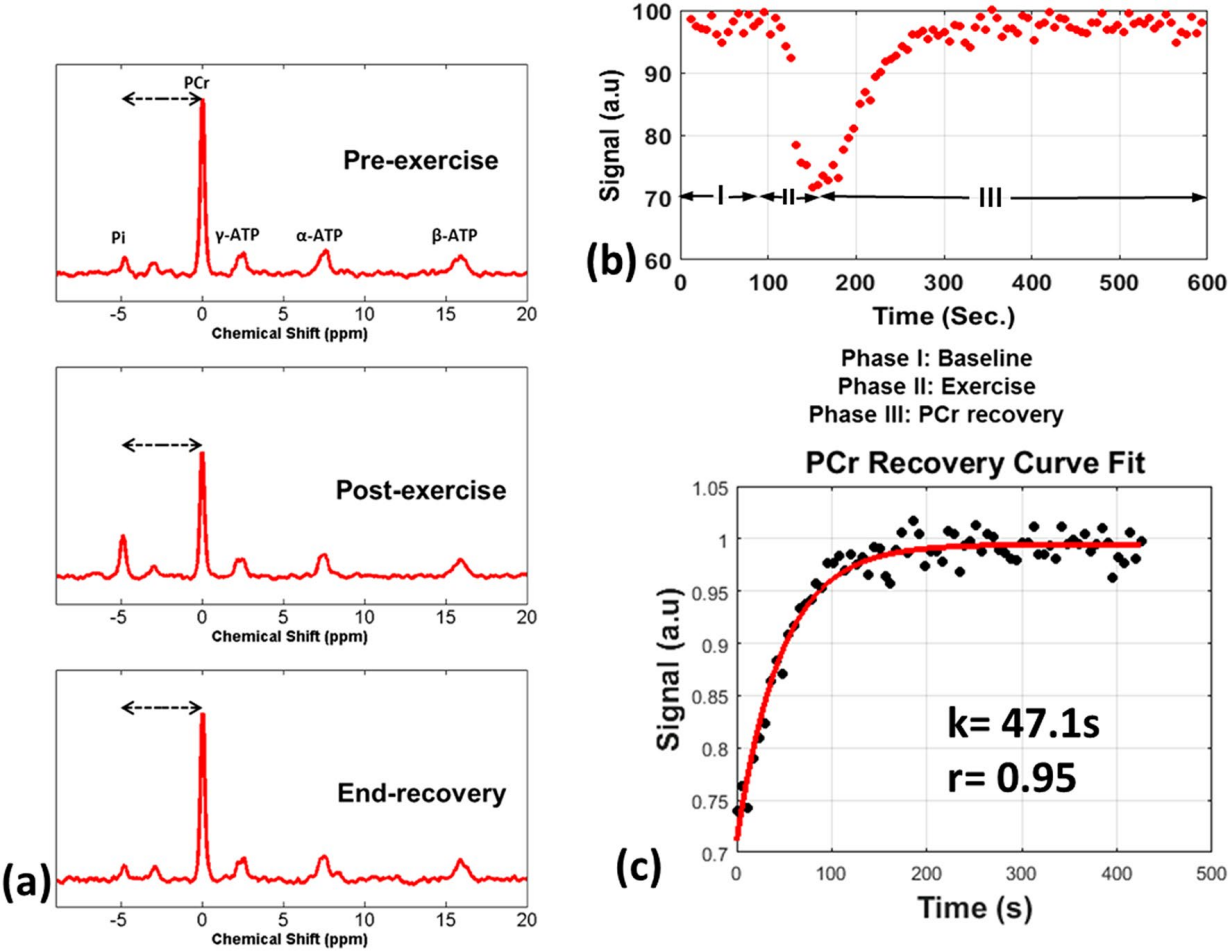
Dynamic ^31^P-MRS results in an HF patient. ^31^P-MRS results in an HF patient (**a**) Shows ^31^P MR spectra from a representative HF patient’s calf muscle pre-exercise, post-exercise, and at end-recovery. The dashed arrows indicate the distance between Pi and PCr peaks (**b**) shows the kinetics of PCr signal intensity during the three phases for the HF patient and (**c**) shows the PCr recovery time constant (*k* = 47.1 s for this patient) after fitting the phase III PCr recovery data.

Figure 2 shows the results obtained from representative ^31^P-MRS spectra in HF patients. Figure 2a shows calf muscle spectra from pre-exercise, post-exercise, and at the end of the recovery period. The HF patients demonstrated an increased rate of PCr depletion during exercise and a slower PCr resynthesis rate post-exercise. The cohort of HF patients did not show significant pH changes as measured by the distance between Pi and PCr peaks. Figure 2b shows the PCr amplitude obtained across time for phase I, II and III in the HF patients. Figure 2c shows Eq. 1 fitted to the phase III PCr recovery data. The PCr recovery rate constant was significantly longer in the HF patient cohort with a mean and standard deviation of 50.1 ± 8.51 s (*P* = 0.0003)..

**Figure 3:**
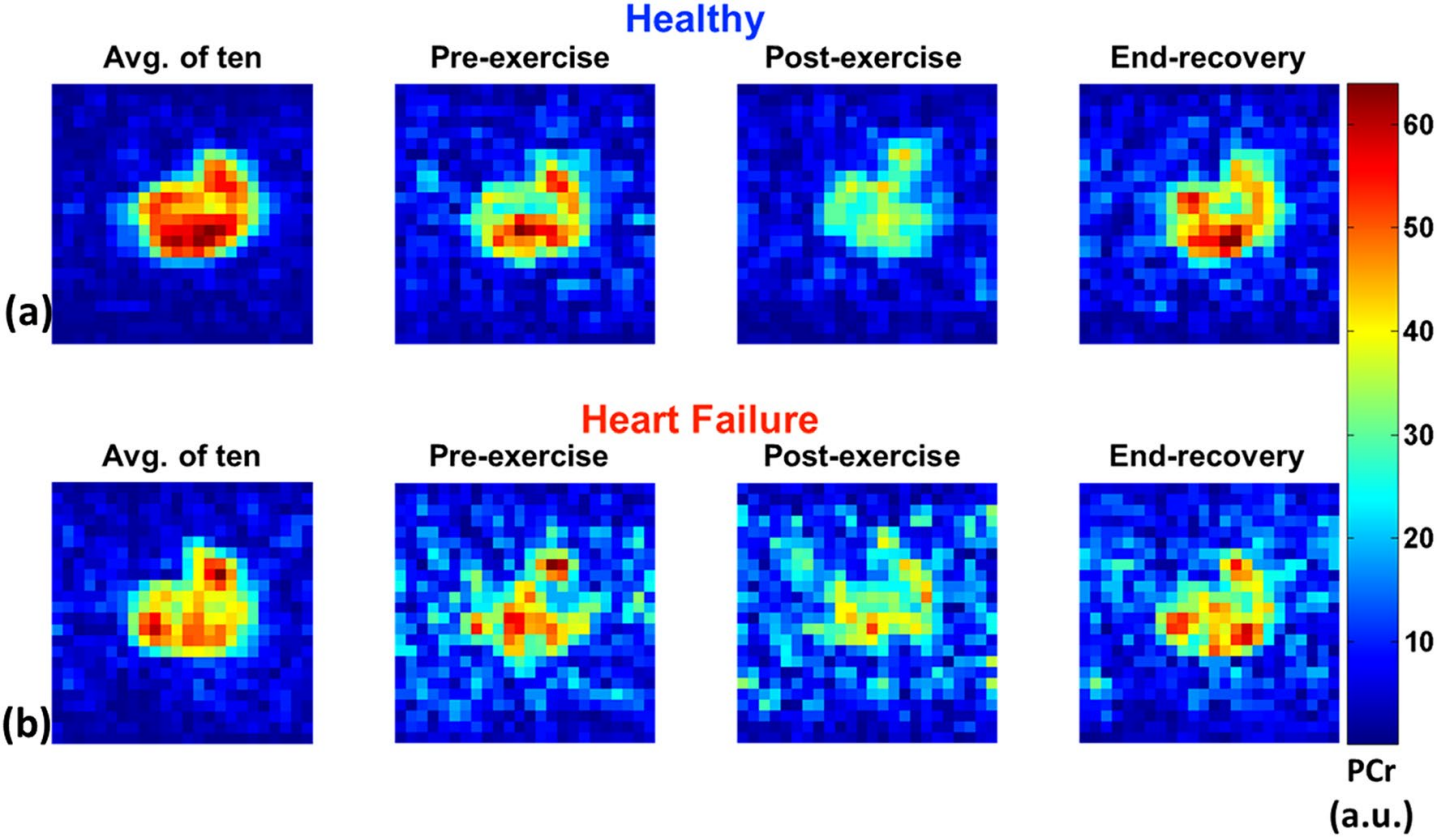
PCr imaging. Representative cross-sectional PCr image of the calf muscle from a volunteer (top) and a HF-patient (bottom), obtained from pre, after exercise and end of recovery respectively. The low SNR of HF-patient image is due to the low PCr concentration of the calf muscles compared to volunteer. The first column shows images with 10 averages to increase SNR.

Representative results from ^31^P MRI images from stage I, II and III in the healthy cohort (a) and the HF cohort (b) are shown in Fig. 3. To boost SNR the first column in the figure shows the average of 10 measurements at end recovery, which shows the difference in PCr resynthesis deficits in HF patients at end-recovery. Columns 2, 3 and 4 show pre-exercise, post-exercise and end-recovery ^31^P MRI images, clearly demonstrating the deficits in PCr resynthesis in the HF patient group. Lower SNR is observed in the HF patient group images, possibly due to greater fatty infiltration in the HF patients.

**Figure 4:**
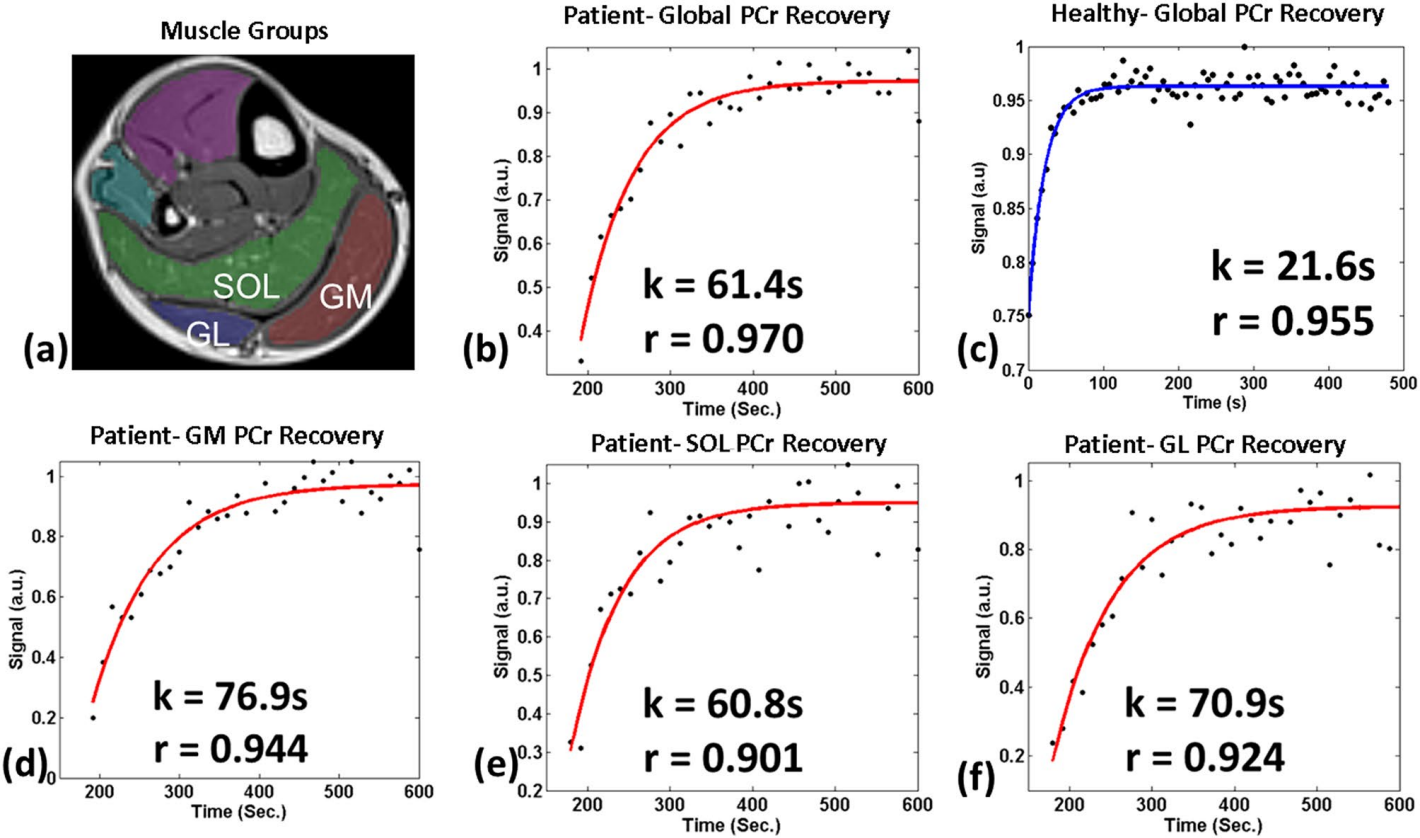
Global and regional PCr recovery using PCr imaging. (**a**) shows an image of calf muscle for anatomical reference and the muscle ROIs drawn for soleus (SOL), gastrocnemius medial (GM) and gastrocnemius lateral (GL). Sub-figures (**b**) and (**c**) show global and regional PCr recovery time constraints assessed using ^31^P-MRI data from a representative HF patient and healthy volunteer by fitting phase III data to Eq. 1. The PCr recovery time constants are significantly delayed (~ 60–70 s) compared to healthy volunteers (~ 20- 25 s). The PCr recovery time in specific muscles (d) GM (e) SOL and (f) GL shows increased depletion and slower recovery in GM and GL muscle groups, suggesting glycolytic metabolism from type II fibers’.

Figure 4 shows representative PCr resynthesis results from ^31^P-MRI from an HF patient, and from a healthy volunteer. Figure 4a shows the manually segmented muscle groups labeled as SOL (soleus), GM (gastrocnemius medial) and GL (gastrocnemius lateral). The global PCr resynthesis rate constant in the healthy subjects with ^31^P-MRI was 25.9 ± 5 s, and that for HF patients was 51.3 ± 8 s. There was some variability in the regional PCr recovery rate constants which ranged from 25.8 ± 4 s to 29.4 ± 4 s in healthy volunteers to 56.1 ± 9 s and 63.5 ± 10 s for HF patients.

**Figure 5:**
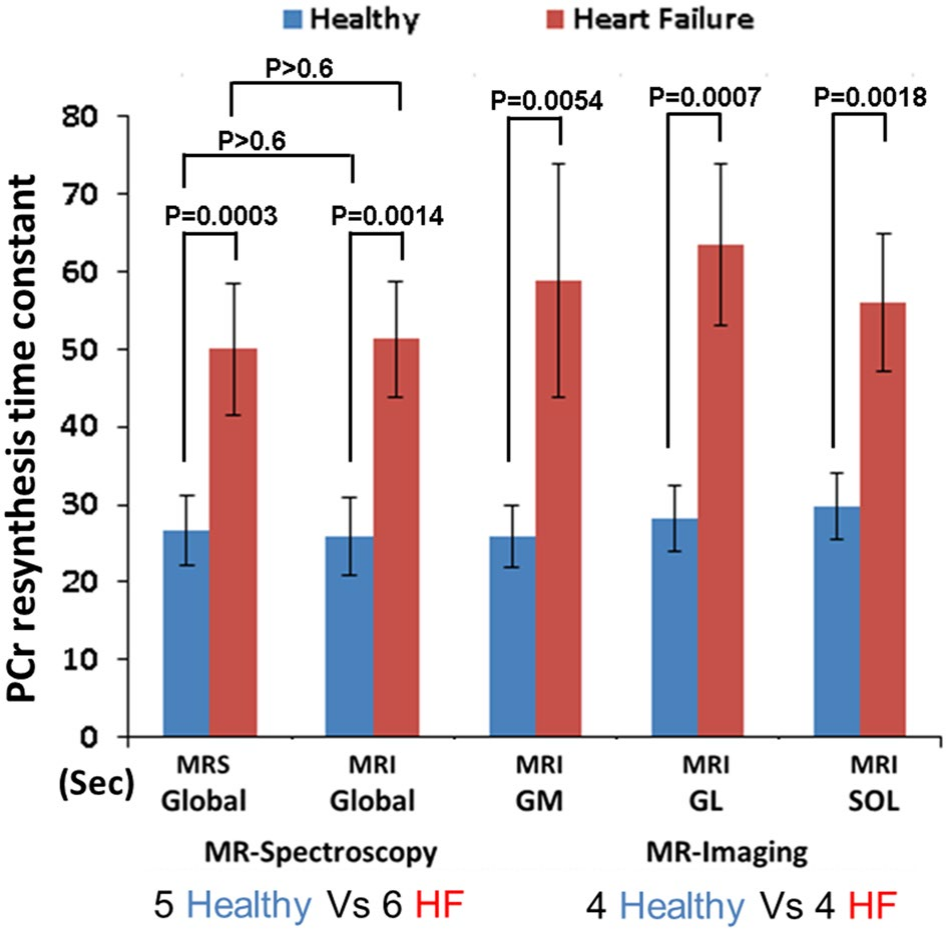
Comparison of results between cohorts. The PCr resynthesis time constant (mean ± SD) between healthy volunteers and HF-patients of Global (^31^P-MRS and MRI) as well as regional (^31^P-MRI) in gastrocnemius medial (GM), gastrocnemius lateral (GL), and soleus muscle (SOL). Significant differences were found in all analysis between volunteers and HF-patients, while two methods globally agreed very well.

Comparison of PCr resynthesis rates between the HF and control groups using ^31^P-MRS and ^31^P-MRI is shown in Fig. 5. A significant difference is observed between HF and control group using unlocalized global MRS measurements (P = 0.0006), and using ^31^P-MRI technique for global measurements (*P* = 0.0014). The measurements using MRS and MRI being comparable did not show significant differences between them (*P* > 0.6). ^31^P MRI technique allows further localization of the PCr resynthesis rates, in the GM, GL and SOL muscle groups. Significant differences in PCr resynthesis were observed in all three muscle groups measured, with GM (*P* = 0.0054), GL (*P* = 0.0007) and SOL (*P* = 0.0018).

**Figure 6:**
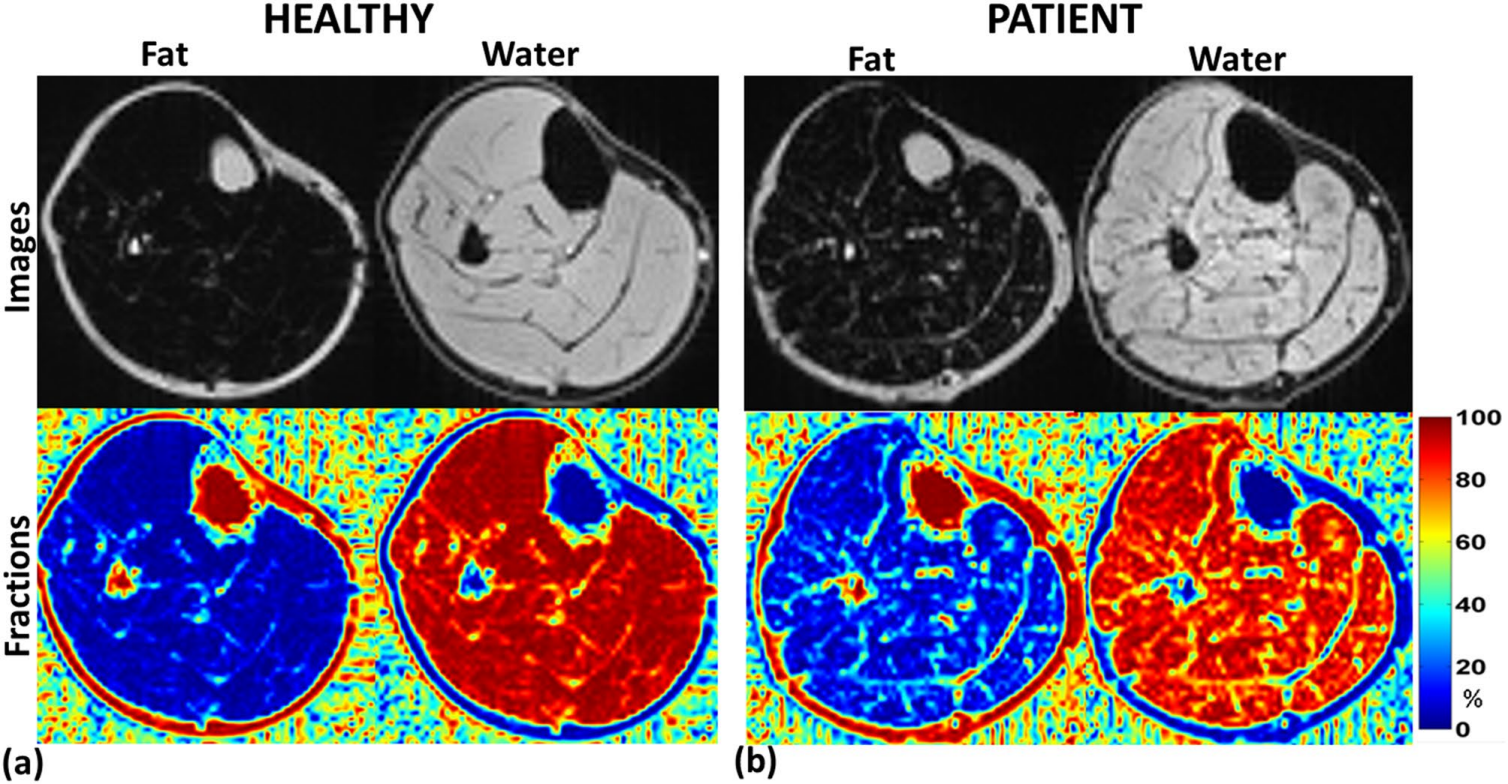
Water-fat Imaging using multi-point Dixon technique. (**a**) shows water-fat imaging results after processing in a healthy volunteer. The first column shows fat image, and the fat fraction in the muscle, and the second column shows the water image and corresponding water fraction. (**b**) shows water-fat imaging results after processing in an HF patient, with the first column showing fat image and fat fractions, while the second column shows the proton image and water fraction. HF patients show considerably increased fatty infiltration in the muscle.

Figure 6 shows multi-echo Dixon fat–water imaging results investigating fatty infiltration. Although, according to the clinical reports of the HF patients, the muscle mass of HF patients was within normal ranges, the fat–water composition showed considerable fatty infiltration in the HF patients compared to healthy controls. Figure 6a shows fat and water images and the corresponding calculated voxel-wise fat-fraction and water-fraction maps in a healthy volunteer. The Fig. 6b shows fat, water images, and fat-fraction and water-fraction maps in an HF patient.

Quantification of parameters from the ^31^P-MRS and Dixon water-fat imaging is summarized in Table 1. The pH in the rest and exercise condition in controls and HF patients does not show a significant change from rest to exercise in both cohorts (*P* = 0.394), while PCr depletion is significantly higher in the HF patient group (*P* = 0.036). Metabolite ratios for PCr/ATP ratio, Pi/PCr ratio and PDE/PCr ratio are shown for different groups and conditions. While considerable differences exist between the groups, they do not reach the level of significance from statistical analysis. The fat fractions in different muscle groups in controls and HF patients are shown. While there is considerably increased fat fraction in HF patients, the differences are not significant.

**Table 1:** Quantification and comparison of the subject cohorts between rest and exercise from ^31^P-MRS and fat fraction.

	Controls		HF Patients (N = 6)		*P*-value
	Rest	Exercise	Rest	Excercise	
pH	7.03 ± 0.04	7.06 ± 0.08	7.01 ± 0.03	7.07 ± 0.07	**0.394**
PCr Depletion (%)	17.8 ± 2.48		35 ± 15.82		**0.036**
PCr Recovery Time constant(s)	26.73 ± 4.49		50.1 ± 8.51		**0.00036**
PCr/ATP Ratio	7.93 ± 1.7	6.43 ± 1.08	8.43 ± 1.39	5.65 ± 1.81	0.083
Pi/PCr Ratio	0.1 ± 0.02	0.26 ± 0.11	0.12 ± 0.03	0.44 ± 0.23	0.066
PDE/PCr Ratio	0.05 ± 0.03	0.05 ± 0.03	0.09 ± 0.05	0.1 ± 0.03	0.082
Fat Fractions (%)					
GM	8.58 ± 4.17		9.46 ± 2.89		0.28
GL	7.17 ± 2.33		8.91 ± 2.66		0.147
SOL	8.53 ± 2.97		10.89 ± 2.56		0.082
AC	5.36 ± 2.00		8.44 ± 0.77		0.082
LC	10.08 ± 3.34		11.94 ± 1.02		0.18

The table shows quantified parameters between controls and the HF patient cohort for change between rest and exercise conditions. The *P*-value calculated is a one-tailed *T*-test, with a significance threshold of 0.05. The pH variation between rest and exercise for both cohorts was not significant (*P* = 0.394), while the PCr depletion in the HF patients following exercise was significantly higher (*P* = 0.036). Bold font highlights important results.

## Discussion

In this study, we showed that ^31^P imaging allows simultaneous measurement of PCr resynthesis rates in several distinct muscles in the post-exercise period to determine the heterogeneities present among different muscle types. The results compare well with ^31^P-MRS studies done on the same subjects. While more studies are warranted to utilize this, the results here suggest that ^31^P-MRI and ^31^P-MRS for lower leg muscle oxidative metabolism may be a potentially useful clinical imaging biomarker for staging and monitoring therapies in HF-patients.

This study used ^31^P-MRS to compare the results obtained from ^31^P imaging. The use of MRS has some unique advantages including being able to look at a range of metabolites that may be of additional interest in HF applications, such as ATP changes, PCr/ATP ratio, Pi/PCr ratio, and phosphomonoesters (PME)/PCr ratio^26^. The calculation of pH from the PCr and Pi peaks is also a very useful measure for some applications, although in this study we used low impact plantar flexion exercise that would prevent acidosis and any pH changes. Some ^31^P-MRS studies that performed submaximal exercise in patients with HF, and with patients with preserved ejection fraction have reported reduced type I (slow twitch) fibers and lower mitochondrial content^27,28^. This study used rapid 3D-TSE-imaging of PCr to assess the variation of the metabolite with exercise in controls and HF patients. Previous studies have shown the feasibility of PCr imaging in controls with similar results^23^, and it has been implemented for assessment using compressed sensing reconstruction and at 7T^24,25^.

Most studies use unlocalized MRS for the measurement of PCr resynthesis following exercise. Localized MRS has also been tested for such applications previously^29^. One study compared the differences between localized and unlocalized PCr resynthesis following exercise showed increased accuracy in PCr resynthesis calculations^30^. In that study, the voxels were placed superficially within a specific muscle group using a surface coil and a large average voxel volume was used (40.4 mL) to increase SNR. The 3D-PCr imaging technique using spectral selectivity used here used a volume coil, which has uniform excitation and signal distribution. This allows uniform signal from the entire muscle group of interest and allows the possibility of assessing bioenergetics of individual specific muscle groups. While the SNR is lower than a surface coil, adequate SNR was used to perform imaging with smaller voxel sizes (4.2 mL at 3 T, 1.6 mL at 7T^25^). In order to achieve adequate spatial–temporal resolution for dynamic ^31^P imaging, the voxel sizes are chosen so as to allow sufficient SNR to measure the PCr metabolite. SNR can be improved with additional averages, but is constrained by reasonable scan times and exercise considerations.

These data with PCr imaging provide some preliminary insights into the mechanistic aspects of oxidative metabolism. Using PCr imaging we are able to see the PCr kinetics in specific muscle groups like the soleus, and gastrocnemius (medial and lateral). Calf muscles have composite amounts of fiber types, with the soleus muscle reported to have ~ 80% slow twitch (Type I) fibers, and the gastrocnemius having ~ 50% fibers of Type I and Type II each^31^. It has been posited that with HF, abnormal skeletal muscle metabolism resulting in an earlier shift to glycolytic metabolism occurs. The studies show an increased utilization of high energy phosphates, increased Pi deposition, earlier acidosis and delayed PCr recovery^14,32,33^. The PCr imaging gives more granular insights about the nature of metabolic activity in specific muscle groups. In this study, the gastrocnemius muscles (GM, GL) in HF patients not only exhibited greater PCr depletion (~ 80%) compared to SOL (~ 70%) after exercise, but delayed PCr recovery occurs in the GM and GL muscle groups suggesting that in HF patients the type II fibers in the GM muscle groups are utilized in a greater proportion. The increased PCr depletion and delayed recovery suggest that GM and GL muscle groups have impaired oxidative metabolism^12^. While conclusive results may not be drawn from these results, the ability of PCr imaging to simultaneously image the whole muscle during an exercise task to explore PCr depletion and recovery in each muscle group can potentially be a powerful approach to stage HF or to monitor treatments. Further studies would be required to investigate the sensitivity of PCr imaging to distinguish deficits between muscle groups in patients.

In this study, the global MRS based PCr resynthesis and global PCr imaging resynthesis in HF patients were significantly slower compared to controls. These results are consistent with a number of previous studies^33–35^. However, it must be noted that good quality PCr imaging results were obtained in only 4 of the 6 HF patients and in 4 of the 5 healthy volunteers representing a 72% success rate, while ^31^P-MRS gave good quality data in all subjects. Further improvements in our technique will increase the robustness of PCr imaging using this technique. Increased fatty infiltration was noted in the HF patients compared to healthy controls. Weiss et al. reported significant fatty infiltration in HF patients, with the infiltration correlated to HF severity, and other comorbid conditions^3^. In the present study, the HF patient cohort did not include severely ill patients.

The incidence of HF is strongly dependent on age, and is a leading cause of morbidity and mortality in elderly persons^36^. The HF cohort in this study was significantly older than the control cohort, with a mean age difference of 20 years. In addition, normal aging induces a shift in skeletal muscle metabolism in the form of sarcopenia^37,38^, the effect of which may be a contributor to the results seen here given the age difference between the cohorts. Increasing age of the HF cohort increases the likelihood of multiple co-morbidities including hypertension and coronary heart disease. Many of the comorbidity factors combined with the effects of aging lead to frailty, muscle atrophy, and decreased functional capacity making diagnosis and staging of the effects of HF in elderly patients challenging.

The study protocol included two exercise instructions during one imaging session, the first for ^31^P-MRS and the second for ^31^P-MRI. While care was taken to not to induce pH changes due to the exercise for the first and second bout of exercise, the order of the MRS and MRI experiments was not varied. Here we assume that the time between the two exercise bouts is sufficient for complete recovery of the calf muscles in both patient groups, as indicated from the lack of change in pH. However, for sicker HF patients, it may not be feasible to perform two bouts of exercise without changing the pH.

The study used a limited cohort of HF patients and controls to demonstrate the utility of PCr imaging for this patient population. A larger study with a more heterogenous cohort in the patient and control population is warranted. In this study, the HF patients recruited were stable class II/III patients. Inclusion of class IV HF patients would give further insights into metabolic deficits at end stages of the disease, and mortality. The temporal resolution of the PCr imaging was 12 s, which is longer than ^31^P-MRS studies. While the results obtained from the MRS and MRI techniques are not significantly different, accelerating data acquisition can result in a comparable temporal resolution to MRS for dynamic studies.

In summary, the results shown suggest that ^31^P-MRS and ^31^P-MRI provide complementary information and may be used quantifying for lower leg muscle oxidative metabolism in HF patients, with ^31^P-MRI allowing further localization of oxidative metabolism. Upon further validation, ^31^P-MRI techniques may be a potentially useful imaging biomarker for staging and monitoring therapies in HF-patients, either in tandem with ^31^P-MRS or as an independent marker for PCr recovery.

## Methods

### Subjects

Six patients (4 male, 2 female, mean age = 56 ± 7 years) with HF, classified as class II or III according to New York Heart Association were included in the present study. All patients were stable, with a clinical diagnosis of chronic HF during the time of the MRI study. Table 2 describes the clinical characteristics of the patient cohort. The control arm of the study consisted of 5 healthy volunteers (all male, mean age = 35 ± 7 years) with no history of hypertension, diabetes mellitus, heart or vascular disease. The study protocol was approved by New York University institutional Internal Review Board (IRB) and all methods were performed in accordance with the IRB guidelines and regulations. All subjects provided written consent prior to participation.

**Table 2:** Patient characteristics.

Patient characteristics	
Age (years)	56 ± 7
BMI (kg/m2)	29 ± 6
LVEF (%)	30 ± 15
BP (mmHg), (Systolic/Diastolic)	121 ± 20/72 ± 12
Creatinine (mg/dL)	0.95 ± 0.27
Hemoglobin (gm/dL)	14 ± 1.4
Ferritin (ng/dL)	128 ± 70

Characteristics of the HF patient cohort are shown in the table in the form of mean ± standard deviation.

### Exercise protocol

Plantar flexion exercises were performed using a custom built MR compatible ergometer^39^. An exercise protocol was designed to ensure enough PCr depletion while keeping the pH change minimal. The subject was in the supine position with the subject’s right calf strapped onto the ergometer, with the middle portion of the calf set as the coil and magnet centers. Before exercise, 1–2 min of data were collected as baseline reference. The subject performed plantar flexions for 1 min to an acoustic cue (0 to 25°) with interval of 1.5 s. This was followed by a recovery phase for the remaining time of the 10-min scan. Data was collected during the entire duration of the exercise protocol. A fixed resistance load using rubber tubing was set to 40% of the subjects’ maximum voluntary contraction. Two exercise sessions were performed with MRS data acquired during the first and MRI data acquired during the second session, with a 10 min rest period in between sessions. Both healthy and HF groups underwent the same protocol.

### MR imaging protocol

This imaging study was performed on a clinical 3 T MRI scanner with multi-nuclear capabilities (Prisma, Siemens Healthineers, Erlangen, Germany). For MRS/MRI measurements a dual tuned ^31^P/^1^H quadrature volume knee coil with inner diameter = 22 cm (Rapid MR, Ohio, USA) was used. The imaging protocol consisted of ^31^P-MRS acquisition during plantar flexion exercise and recovery, and ^31^P-MRI imaging performed during a second plantar flexion exercise and recovery. Proton based Dixon water-fat imaging was performed at rest to quantify fatty infiltration in the cohorts.

### Dynamic ^31^P-MRS protocol

^31^P-MRS data were continuously collected during a pre-exercise period, a plantar flexion exercise and a recovery period. A vendor supplied unlocalized free induction decay (FID) sequence was used to acquire ^31^P-MRS data, after the target volume was shimmed. The acquisition TR was 6 s and 2048 points with a spectral width of 3 kHz were acquired. In total, 100 FID measurements were acquired in 10 min, resulting in a temporal resolution of 6 s.

### Dynamic ^31^P-MRI protocol

To perform ^31^P-MR imaging, the subject performed a second exercise task. A modified version of centric 3D-turbo spin echo (TSE) sequence was used that allowed spectral selectivity of the PCr peak, which allowed acquisition of two partitions per TR^40^. Parameters were as follows: echo train length (ETL) = 24; TE and echo-spacing = 26 ms; acquisition bandwidth = 1.6 kHz; matrix size = 24 × 24 × 4; FOV = 220 × 220 × 200 mm (voxel size = 4.2 mL); TR = 6 s, resulting in 12 s temporal resolution and 50 measurements in 10 min. A 16 ms spectral selective Gaussian pulse was used for excitation of PCr.

### Dixon water-fat imaging

Proton based Dixon multi-echo imaging was used for water-fat quantification. The multi-echo GRE sequence parameters included TR = 9.3 ms, TE = 2.26, 3.08, and 3.90 ms, FOV = 180 mm, matrix size = 128 × 128, resolution = 1.4 × 1.4 mm^2^, total acquisition time = 4.5 min.

### Data analysis

All ^31^P-MR spectra were post-processed via zero-filling to 8192 points. Zero and first order phase correction and baseline correction were performed on the spectra. After normalizing the MRS and MRI (mean signal intensity of all voxels within muscle) data to the mean pre-exercise value for each subject, a mono exponential recovery function was fitted to the data, using a least squares minimization algorithm.

1 $$ PCr\left( t \right) \, = \, PCr_{0} + \, C\left( {1 \, - \, e^{ - t/k} } \right) $$

In Eq. (1), PCr_0_ is the PCr level at the end of exercise, C is the difference between the steady-state level and the PCr after recovery, and k is the time constant (1/rate constant) of PCr resynthesis in seconds. For the ^31^P-MR images manual segmentation of the different muscles (GM, GL and SOL) was done. The data was fitted to Eq. (1) in each volume of interest in order to measure the regional time constant of PCr resynthesis.

Iterative decomposition of water and fat with echo asymmetry and least-squares estimation (IDEAL) technique^41^ was used to separate the water and fat images in the multi-echo Dixon data. The water and fat fraction images were calculated from the separate water and fat images for each voxel.

### Statistical analysis

The mean values and standard deviations of the PCr resynthesis rate was calculated for each ROI from the ^31^P imaging technique. The unpaired t-test was used to compare the results from patient and control cohorts with the unlocalized ^31^P-MRS, ^31^P-MRI, and fatty infiltration quantification. A P-value of less than 0.05 was used for analysis as threshold for significance.

## Data Availability

The datasets generated and analyzed during the current study are available from the corresponding author on reasonable request.
